# Viral and bacterial upper respiratory tract infection in hospital health care workers over time and association with symptoms

**DOI:** 10.1186/s12879-017-2649-5

**Published:** 2017-08-09

**Authors:** C. Raina MacIntyre, Abrar Ahmad Chughtai, Yi Zhang, Holly Seale, Peng Yang, Joshua Chen, Yang Pan, Daitao Zhang, Quanyi Wang

**Affiliations:** 1 School of Public Health and Community Medicine, UNSW Medicine, University of New South Wales, Level 2, Samuels Building, Sydney, 2052 Australia; 2 College of Public Service & Community Solutions, Arizona State University, Phoenix, AZ USA; 3 The Beijing Centre for Disease Prevention and Control, Beijing, China

**Keywords:** Bacterial colonisation, Masks, Respiratory infections, Infection control

## Abstract

**Background:**

Bacterial colonisation of the respiratory tract is commonly described and usually thought to be of no clinical significance. The aim of this study was to examine the presence and significance of bacteria and viruses in the upper respiratory tract of healthcare workers (HCWs), and association with respiratory symptoms.

**Methods:**

A prospective cohort study was conducted in China and 223 HCWs were recruited from fever clinics and respiratory, paediatric, emergency/Intensive medication wards. Participants were followed over 4 weeks (7th May 2015 to 4th June 2015) for development of clinical respiratory illness (CRI). Nasopharyngeal swabs were obtained at baseline and at the end of the study. The primary endpoints were laboratory-confirmed bacterial colonisation and viral respiratory infection. Rates of the following infections in symptomatic and asymptomatic participants were compared at the start or end of the study; 1) all bacterial/viral infections, 2) bacterial infection and bacterial-viral co-infections, excluding virus only infections, and 3) only bacterial infections.

**Results:**

Bacterial colonisation was identified in 88% (196/223) of participants at the start or end of the study. Among these participants, 66% (148/223) had only bacterial colonisation while 22% (48/223) had co-infection with a virus. Bacteria were isolated from 170 (76.2%) participants at baseline and 127 (57%) participants at the end of the study. Laboratory confirmed viral infections were identified in 53 (23.8%) participants - 35 (15.7%) at the baseline and 20 (9.0%) at the end of the study.

CRI symptoms were recorded in 12 participants (4.5%) and all had a positive bacterium isolation at baseline (*n* = 11) or end of the study (*n* = 1). Among asymptomatic participants, 187 (87%) had bacterial colonisation or bacterial/viral co-infection at baseline or end of the study. Viruses were also isolated from 5 (2.4%) asymptomatic cases. Rates of all infection outcomes were higher in symptomatic participants, however differences were not statistically significant.

**Conclusion:**

We isolated high rates of bacteria and viruses in the upper respiratory tract of hospital HCWs, which may reflect greater exposure to respiratory infections in the hospital. Although respiratory infections are mostly symptomatic, the association between bacterial colonization and symptomatic illness is not clear. In the healthcare setting, HCWs may acquire and transmit infection to patients and other HCWs around them. Larger studies are required to explore ongoing occupational risk of respiratory infection in hospitals HCWs.

## Background

The significance of finding pathogenic bacteria in the upper respiratory tract is unknown, as it is generally thought that many people are asymptomatic carriers of colonising bacteria [1–3]. To date, there has been very little research looking at the role of upper respiratory tract bacteria as a cause of mild respiratory illness. Healthcare workers (HCWs) are at high risk of nosocomial infection with respiratory, blood borne and other infections [4–6]. Respiratory infections are the leading cause of acute infections in humans. Whilst the burden of disease is well recognised in the elderly and immunocompromised, limited information exists on the impact of respiratory infections on HCWs [6–8]. Nosocomial infections among HCWs result in increased rates of illness, absenteeism and even death amongst HCWs [5, 9] and also comes at a large financial cost to the health care system [10]. HCWs may also be a source of infection in nosocomial outbreaks [11]. Transmission of influenza from patient-to-HCW and HCW-to-patient have been identified in many cases highlighting the importance HCWs play in hospital transmission [12].

Varying degrees of nasopharyngeal (NP) bacterial colonisation are reported in healthy individuals, depending on the age of participants, site of specimen collection and vaccination status [13, 14]. Certain factors increase the risk of nasopharyngeal carriage such as age, geographical area, vaccination status, immunity and socio-economic status [1, 13–15]. There are limited data from HCWs, and most studies are conducted in children, who generally have higher rates of NP colonisation compared to adults [3, 16]. Commonly isolated organisms are *S*treptoc*occus pneumoniae (pneumococcus), Haemophilus influenzae, Moraxella catarrhalis* and *Staphylococcus aureus* [17]. Testing of healthy children showed 50% NP carriage rate for *S. aureus* [13], 55–69% for *Streptococcus pneumoniae* [13, 14, 18], 67–74%for *Moraxella. catarrhalis* [13, 14] and 57–83% for *Haemophilus influenzae* [13, 14, 18].

In a study of both inpatients and outpatient children the overall swab positivity rates were 31.5% (63/200), with *Streptococcus pneumoniae*, *Haemophilus influenzae* and Group A *Streptococcus* accounting for 22%, 5% and 4.5% respectively [19]. In a study in Italy, the rate of *Streptococcus pneumoniae*, *Haemophilus influenzae, and Moraxella catarrhalis* found 3.5%, 11.9% and 4.1% carriage respectively (overall 17.9%) [20]. In Australian Aboriginal adults, rates of bacterial colonisation were 26%, 23% and 17% for *Streptococcus pneumoniae, Haemophilus influenza a*nd *Moraxella catarrhalis* respectively [14]. Lower carriage rates of *Haemophilus influenzae* (0%) and S*treptococcus* pneumoniae (10.8%) were observed in a study in China [18]. An Australian study also reported very low rate (1/315) of *Streptococcus pneumoniae* in hospitalised elderly patients [21].

The finding of a virus in the upper respiratory tract is generally thought to be clinically significant. However the significance of asymptomatic viral infection has not been explored in existing literature, and viruses detected in the upper respiratory tract are generally assumed to be pathogenic. Some studies have found that up to 1 in 3 subjects with influenza infection may be asymptomatic [22], whilst asymptomatic infection with parainfluenza virus has also been observed [23]. One study also demonstrated shedding of parainfluenza virus from healthy subjects over an extended period of time [24]. Whilst little research exists on asymptomatic viral infections, these cases suggest that viruses can be transmitted by asymptomatic subjects unknowingly. A better understanding of the role of bacterial and viral infections in HCWs and their association with the respiratory symptoms is warranted. The aim of this study was to examine the presence and significance of bacteria and viruses in the upper respiratory tract of HCWs.

## Methods

A prospective cohort study was conducted in four hospitals in Beijing, China. Participants were hospital HCWs aged between 18 and 65 from respiratory wards, paediatric wards, intensive care unit, outpatient fever clinics (special clinics in Chinese hospitals for management of febrile patients) and emergency units. Participants were followed over a 4 week period and were asked to report any symptoms that developed over the study period. Recruitment formally commenced on the 7th of May 2015 and final follow up was completed on the 4th of June 2015.

### Eligibility

HCWs (doctors and nurses) from the selected wards of four hospitals were invited to participate in the study. Full-time HCWs, aged 18 and over were eligible for participation in the study. Participants were excluded if they: [1] were unable or refused to consent; [2] had a current respiratory illness; rhinitis and/or allergy; [3] work part-time or [4] were not available for the 4 weeks follow-up.

### Recruitment

Information about the study was provided to staff members by district level staff members from the Beijing Centre for Disease Prevention and Control (CDPC). Hospital staffs were invited to attend information sessions, which were held at different times of the day. Information sheets and consent forms were given out during these sessions and interested staff members asked to return the completed forms if they had agreed to give consent and participate in the study. Additional copies of the participant information sheet were also left with designated staff members from each ward to pass onto staff members who were unable to attend the information sessions.

### Data collection and follow up

At time of recruitment, baseline nasopharyngeal swabs were collected by trained staff and tested in the Beijing CDPC laboratory. Detailed demographic and clinical details for all participants were also collected. This included age, sex, smoking history, comorbidities, vaccination status, medications, use of personal protective equipment, performing high risk procedures, antivirals and results of laboratory tests.

At the end of the 4 weeks, another set of nasopharyngeal swabs was collected from all participants. A second (exit) survey was administered at the same time to participants to collect information about the development of any respiratory symptoms in the previous 4 weeks. Clinical respiratory illness (CRI) was defined as two or more respiratory symptoms (cough, nasal congestion, runny nose, sore throat or sneezes) or one respiratory symptom and a systemic symptom (chill, lethargy, loss of appetite, abdominal pain, muscle or joint aches).

The primary endpoints were:

1. Laboratory-confirmed bacterial colonisation in symptomatic/non-symptomatic subjects. Multiplex PCR was used to test for *Streptococcus pneumoniae, Legionella, Bordetella pertussis, chlamydia pneumoniae, Mycoplasma pneumoniae* or *Haemophilus influenzae type B* (Seegen, Inc., Seoul, Korea).
2. Laboratory-confirmed viral respiratory infection in symptomatic/non-symptomatic subjects, defined as detection of *Adenoviruses, Human metapneumovirus, Coronaviruses 229E/NL63 and OC43/HKU1, Parainfluenzaviruses 1, 2 and 3, Influenza viruses A and B, Respiratory syncytial viruses A and B, or Rhinoviruses A/B* by nucleic acid testing (NAT) using a commercial multiplex polymerase chain reaction (PCR) (Seegen, Inc., Seoul, Korea) [25].

### Specimen collection and testing

Double rayon-tipped, plastic-shafted swabs were used to scratch both tonsilar areas and the posterior pharyngeal wall of participants. These samples were then transported immediately after collection to the Beijing CDPC laboratories, or stored at 4 °C for up to 48 h if transport is delayed. Viral DNA/RNA was extracted from each respiratory specimen using the Viral Gene-spinTM Kit (iNtRON Biotechnology, Inc., Seoul, Korea) according to the manufacturer’s instructions. Reverse transcription was performed using the RevertAidTM First Strand cDNA Synthesis Kit (Fermentas, ON, Canada) to synthesise cDNA. Multiplex PCR was carried out using the Seeplex® RV12 Detection Kit (Seegen, Inc., Seoul, Korea). A mixture of clones of the 12 viruses tested was used as a positive control template, and sterile deionised water was used as a negative control. Viral isolation by MDCK cell culture was undertaken for some of the influenza samples that are influenza NAT positive. NAT using a multiplex PCR was also done on the same DNA/RNA extract as used for the viral PCR (Seegen, Inc., Seoul, Korea). Specimen processing, DNA/RNA extraction, PCR amplification, and PCR product analyses were conducted in different rooms to avoid cross-contamination.

### Analysis

Rates of bacterial, viral and co-infections were measured at the start and end of the study period and were also compared in symptomatic and non-symptomatic participants. We performed analysis considering three outcomes. First we compared rates of all bacterial infection at the start or end of the study including bacterial/viral co-infections, in symptomatic and asymptomatic participants. Then we compared rates of bacterial infection and co-infection in symptomatic and asymptomatic participants - five cases with viral only infections were excluded from this analysis. Finally we compared rates of only bacterial infection excluding viral co-infection, in symptomatic and asymptomatic participants.

The primary endpoints of interest were analysed by binary logistic regression models. Univariate analyses were performed for main exposure variable and other variables such as age, sex, occupation, vaccination status, performing high-risk procedures, mask use, smoking status, pre-existing illness, respiratory symptoms within the family and ward type. Multivariate analyses were also performed and all variable were included in the model. The data were analysed using SAS v. 9.4 (SAS Institute Inc., USA).

### Sample size

Sample size was based on 95% confidence and 80% power to detect difference between rates of bacterial colonisation in symptomatic and asymptomatic individuals, if we assume 20% of HCWs will have bacterial colonization. These estimates are based on previous studies that describe adult colonisation rates. The sample size was calculated in Epi Info 2000 [26]. In order to allow for loss to follow up, 220 hospital staff members were to be recruited.

### Ethics approval

The study protocol was approved by the Human Research Ethics Committee of the Beijing Ministry for Health and HREC University of New South Wales (HC14325).

## Results

A total of 223 participants were recruited from four hospitals and followed for 4 weeks. Most participants were female (84.3%), with a graduate degree (71.3%) and were not vaccinated for influenza (78.5%) during the study season (Table 1). The mean age of participants was 36.7 years (SD ±9.7 and range 20–65 years) and around half of them were doctors. Thirteen percent (29/223) of participants had at least one pre-existing medical condition and 64% (143/223) had performed high risk procedures during the study period.

**Table 1:** Demographic characteristics of participants (*n* = 223)

Variable	Number	Precent/mean SD
Gender		
Male	35	15.7
Female	188	84.3
Age		36.7 (± 9.7 SD)
Profession		
Doctor	104	46.6
Nurses	119	53.4
Education		
Undergraduate	37	16.6
Graduate	159	71.3
Post graduate	27	12.1
Smoking status		
Current/ex-smoker	11	4.9
Never	212	95.1
Influenza vaccine		
Yes	48	21.5
No	175	78.5
Medical conditions^a^		
Yes	29	13.0
No	194	87.0
High risk procedures^b^		
Yes	143	64.1
No	80	35.9
Ward		
Respiratory	99	44.4
Paediatric	47	21.1
Fever clinics	31	13.9
Emergency/ Intensive medication^c^	46	20.6
Hospital		
A	50	22.4
B	55	24.7
C	57	25.6
D	61	27.4

^a^Include asthma, diabetes, immunosuppression and other

^b^Include suctioning of airways, endotracheal intubation, sputum induction, chest, physiotherapy bronchoscopy

^c^45 cases in emergency and 1 in intensive medication unit

**Fig. 1:**
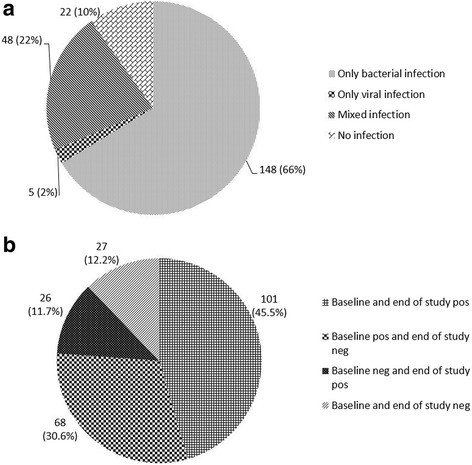
**a** Rates of bacterial, viral and co-infections; **b** Bacterial colonisation at baseline and at end of the study

Bacteria were isolated from 170 (76.2%) participants at baseline and 127 (57%) participants at the end of the study (Table 2). If co-infections were excluded, bacteria were isolated from 57% participants (128/223) at baseline and 44% (98/223) at end of the study. Overall 196 (88%) participants had bacterial colonisation at start or end of the study - 148 participants (66%) had only bacterial colonisation while 48 (22%) participants had co-infection with a virus (Fig. 1a). Among the total participants, 101 (45.5%) were positive for bacteria at both baseline and end of the study, 68 (30.6%) were positive at baseline and negative at the end, 26 (11.7%) were negative at baseline and positive at the end and 27 (12.2%) were negative at both periods (Fig. 1b). Among all bacterial positive cases, *Streptococcus pneumoniae* (isolated or co-infected with Haemophilus influenza) was the most commonly isolated organism at baseline (96%, 163/170) and end of the study (72%, 91/127). Sixty-seven cases were positive for *Streptococcus pneumoniae* at both baseline and end of the study – 18 from respiratory ward (18%, 18/99), 18 from paediatric ward (38%, 18/47), 9 from fever clinics (29%, 9/31) and 22 from emergency ward (48%, 22/46).

**Table 2:** Laboratory results (*n* = 223)

	Number at the baseline^a^	%	Number at the end of study^a^	%
Bacteria isolated				
Streptococcus pneumoniae	98	43.9	38	17
Haemophilus influenzae	7	3.1	36	16.1
Streptococcus pneumoniae and Haemophilus influenzae	65	29.1	53	23.8
*All bacteria* ^c^	*170*	*76.2*	*127* ^b^	*57*
*Only bacteria (no co-infection)*	*128*	*57.4*	*98*	*43.9*
Virus isolated				
Rhinovirus/Enterovirus	24	10.8	10	4.5
Influenza A (H3N2)	6	2.7	5	2.2
Rhinovirus/Enterovirus/Influenza A (H3N2)	1	0.4	0	0
Other^d^	4	1.8	5	2.2
*All virus* ^c^	*35*	*15.7*	*20*	*9.0*
*Only virus (no co-infection)*	*3*	*1.3*	*2*	*0.9*
Co-infection	29	13.0	11	4.9

^a^101 cases were positive at both baseline and end of the study

^b^Including 26 new positive samples which were negative at baseline

^c^One participant was not sampled at end of the study. Among 35 cases at baseline - 14 were from respiratory ward, 6 from paediatric ward, 8 from fever clinics and 7 from emergency ward. Among 20 cases at the end of the study - 7 were from respiratory ward, 8 from paediatric ward, 1 from fever clinics and 4 from emergency ward

^d^At baseline other includes ADV [1], CoV229E [1], CoVC229E [1], Metapneumovirus [1] and at the end of the study other includes ADV [2], CoV229E [1], CoVC229E [1], Metapneumovirus [1]

There were 35 (15.7%) laboratory confirmed viral infections found at baseline and 20 (9.0%) found at the end of the study (Table 2). *Rhinovirus/enterovirus* was the most common viral pathogen accounting for 24 (10.8%) and 10 (4.5%) infections at baseline and the end respectively. Other viruses detected included *Adenovirus, Coronavirus, H1N1 and H3N2 influenza virus* and *human metapneumovirus*. Rates of bacterial/viral co-infections were 13% (29/223) at baseline and 4.9% (11/223) at the end of the study.

Twelve participants (4.5%) developed clinical respiratory illness (CRI) during the 4 week study period and all of these 12 HCWs had positive bacteria isolation at baseline (*n* = 11, including 4 co-infection with a virus) or end of the study (*n* = 1). Among asymptomatic participants, 187 (87%) had bacterial colonisation or co-infection at baseline or end of the study. Viruses were also isolated from 5 (2.4%) of asymptomatic cases (Table 3).

**Table 3:** Rates of bacterial, viral and co-infection among symptomatic and asymptomatic participants

Symptomatic (CRI)	Bacterial colonisation	Viral infection	Co-infection	Negative swab	Total
Yes (12)	8 (66.7%)	0	4 (33.3%)	0	12 (100%)
No (213)	140 (66.4%)	5 (2.4%)	44 (20.9%)	22 (10.4%)	211 (100%)

Rates of bacterial colonisation were compared among symptomatic and non-symptomatic participants in the Table 4. In all three outcomes, rates of bacterial colonisation were higher in symptomatic participants, compared to non-symptomatic, although differences were not statistically significant.

**Table 4:** Comparing rates of bacterial colonisation in symptomatic and non-symptomatic participants

CRI	Bacterial colonisation	Rate (%)	OR	*P* value
All bacteria positive at start or end of the study (including bacteria/viral co-infections) (*n* = 223)				
Yes	12/12	100	3.7 (0.19–72.55)	0.385
No	184/211	87.2	Ref	
All bacteria positive at start or end of the study (excluding virus only infection) (*n* = 218)				
Yes	12/12	100	3.1 (0.16–59.69)	0.463
No	184/206	89.3	Ref	
Only bacterial infection, excluding viral and co-infection infection cases (*n* = 170)				
Yes	8/8	100	2.72 (0.13–57.84)	0.521
No	140/162	86.4		

In univariate analysis, rates of bacterial colonisation were higher (OR 0.31, 95% confidence interval 0.12 to 0.75) in females (90.4%, 170/188) compared to males (74.3%, 26/35) however this was not statistically significant (Table 5). No other variable was associated with bacterial colonisation.

**Table 5:** Univariate and multivariate analysis (*n* = 223)

Variable	Bacterial colonisation	Rate	OR (95% CI)	AOR (95% CI)	*P* value
CRI					
Yes	12/12	100	3.72 (0.19 to 72.55)	3.64 (0.22 to 60.36)	0.367
No	184/211	87.2	Ref	Ref	
Gender					
Male	26/35	74.3	**0.31 (0.12 to 0.75)**	0.33 (0.11 to 1.01)	0.052
Female	170/188	90.4	Ref	Ref	
Age			0.97 (0.93 to 1.02)	0.98 (0.93 to 1.03)	0.549
Profession					
Doctor	89/104	85.6	0.66 (0.30 to 1.49)	0.92 (0.32 to 2.59)	0.869
Nurses	107/119	89.9	Ref	Ref	
Smoking status					
Current/ex-smoker	8/11	72.7	0.34 (0.08 to 1.37)	0.54 (0.12 to 2.42)	0.418
Never	188/212	88.7	Ref	Ref	
Influenza vaccine					
Yes	42/48	87.5	0.95 (0.36 to 2.52)	0.69 (0.26 to 1.86)	0.463
No	154/175	88			
Medical conditions^a^					
Yes	25/29	86.2	0.84 (0.27 to 2.63)	0.77 (0.24 to 2.46)	0.664
No	171/194	88.1	Ref	Ref	
High risk procedures^b^					
Yes	124/143	86.7	0.72 (0.30 to 1.74)	0.72 (0.26 to 2.01)	0.528
No	72/80	88.9	Ref	Ref	
Mask use					
Yes	172/196	87.8	0.90 (0.25 to 3.20)	0.83 (0.24 to 2.82)	0.765
No	24/27	88.9	Ref	Ref	
Ward					
Respiratory	81/99	81.8	Ref	Ref	
Paediatric	44/47	93.6	3.26 (0.91 to 11.68)	2.72 (0.78 to 9.47)	0.117
Fever clinics	29/31	93.5	3.22 (0.70 to 14.75)	2.62 (0.56 to 12.24)	0.221
Emerg/ICU^c^	42/46	91.3	2.33 (0.74 to 7.38)	2.52 (0.77 to 8.24)	0.126

Bold shows significant result

^a^Include asthma, diabetes, immunosuppression and other

^b^Include suctioning of airways, endotracheal intubation, sputum induction, chest, physiotherapy bronchoscopy

^c^45 cases in emergency and 1 in intensive medication unit

## Discussion

We found a very high rate of bacterial colonisation in HCWs, especially *Streptococcus pneumonia*, with fluctuation in infections over a period of weeks. Almost 88% of all HCWs had bacteria detected in the nasopharynx at baseline, the end of the study period or both. This is a much higher rate of colonisation compared to other studies of adults. For example, other studies of adults show rates of 5–20% [27, 28]. We have previously shown only 0.3% of elderly subjects carry pneumococcus in the nasopharynx. The finding of such a high rate in this HCW population may reflect greater exposure to respiratory infections in the hospital setting and confirms the continual, ongoing risk to HCWs in the hospital setting.

Respiratory infections in hospital HCWs are of particular concern due to the risk of transmission to patients who are ill and/or immunocompromised. Respiratory tract infections generally present with symptoms such as fever, tachypnea, shortness of breath and cough. However the relationship of bacterial colonization to symptomatic illness has not been studied extensively. We found a very high and dynamic rate of bacterial colonisation in hospital HCWs, with changes from baseline to the end of the follow up period in the individuals with infection as well as the types of infection.

Colonisation is important as this may progress to invasive disease [1]. Bacterial colonisation may be an important source of horizontal spread of infection within the community [1]. Among 170 HCWs with positive bacterial result at baseline, 68 (40%) became negative at the end of the study. Natural clearance of bacteria in asymptomatic and symptomatic subjects has not yet been studied. The rates of bacterial colonisation in symptomatic HCWs were higher than in asymptomatic HCWs, but this was not significant. Bacterial colonisation in the majority of the HCWs resolved without any treatment or development of symptoms. We found 12 cases of CRI developed over 4 weeks, 11 of which had bacterial colonisation at baseline. If bacterial shedding occurs asymptomatically, then a large amount of undetected transmission may be occurring in hospitals. This may be important for bacteria such as pneumococcus, where the transition from carriage to invasive disease is thought to occur soon after acquisition of infection.

Of interest, we identified 5 cases of asymptomatic viral infection - four *rhinovirus/enterovirus* and one influenza *A(H3N2)*. Few studies have been conducted on the incidence of asymptomatic viral infection, and of these, the results are often inconsistent. One study examined the rate of asymptomatic infection resulting from inoculation and found that 1/3 of participants did not develop any symptoms [23] whereas a more recent study found the rate of respiratory illness attributable to influenza infection to be 27 respiratory illnesses per 100 persons [29]. Our findings indicated a high rate of asymptomatic infection at baseline, being cleared without the development of symptoms. The clinical significance of such findings is still unknown with limited information on viral shedding and transmission in asymptomatic subjects. It is well known that influenza virus is shed from the respiratory tract in the incubation period in asymptomatic subjects, and asymptomatic infection has also been observed with parainfluenza virus infection [22]. It has also been found that viral shedding of influenza occurs on average for 5 days after infection, indicating that some positive tests could have been in HCWs recovering from influenza [22]. Asymptomatic viral infections pose a significant risk of nosocomial transmission to both patients and HCWs.

We found many co-infections in this study. Previous studies have demonstrated that a viral infection may facilitate bacterial colonisation or co-infection with *S. pneumoniae* [30]. This may be a significant concern as such co-infection has been associated with significantly higher morbidity and mortality [31]. A growing body of evidence suggests that the risk of bacterial respiratory infections is increased by co-infection with viruses and vice-versa, however bacterial respiratory tract infections are generally not considered a major occupational hazard. Despite documented outbreaks of *Bordetella pertussis, Chlamydia pneumoniae* and *Mycoplasma pneumoniae* [32–36], there are few prospective studies of bacterial respiratory infections or colonization, nor consideration of the clinical implications for HCWs. The risk of co-infection has been reported in schools and daycare centres with subsequent community transmission [3], but not in HCWs. It has also been suggested that viral infection may facilitate bacterial colonisation of the respiratory tract particularly with *S. pneumoniae*. Studies in mice have found that *influenza virus* infection increases the transmission and burden of pneumococcal disease [30]. Similar findings have been reported in other studies demonstrating significantly higher morbidity and mortality of cases with influenza virus co-infection with S. pneumoniae [37]. This is suggestive that the role and significance of viral infection in the nasopharynx may be complex, highlighting the need for further research into this topic.

Being a healthcare provider has been identified as a major risk factor for respiratory infections [38, 39], however even within HCWs, the risk varies significantly. Hand hygiene, use of personal protective equipment (PPE) and working on intensive care units (ICUs) have been associated with risk of influenza [40]. Interestingly, factors such as vaccination status, performing high-risk procedures, working on respiratory and paediatric wards and smoking were not found to be significant in predicting bacterial colonisation in this study. Smoking, influenza vaccination status and ward type in hospitals have been previously identified as risk factors for respiratory infection in various groups [40, 41] however our findings suggest that such risk factors may not be absolute and may vary in different situations. The effect of vaccination also needs to be studied. Some studies show that pneumococcal vaccination may reduce colonisation with vaccine-serotype pneumococcal infection, though replacement by other strains reduces the overall effect [1]. Previous studies showed that medical masks and respirators reduce the risk of bacterial respiratory infections [42], which further supports the occurrence of nosocomial transmission of bacteria.

The limitations of this study include that we did not test for bacterial or viral infection at the time of reported symptoms. This would confirm that an infection was the cause of symptom development and also ensure that no other infections were missed within the 4 weeks. Our sample size may have also been too small to detect differences in colonisation between symptomatic and asymptomatic subjects, or for analysis of risk factors such as smoking and underlying disease as there were very few participants in these categories. We were unable to recruit the initially planned sample size so a larger scale study is warranted. The selected follow up period of 4 weeks was the maximal period of follow up possible within the available resources for the study, but longer follow up would be valuable. Finally, these results may not be generalised due to varying geographical distribution of pathogens and vaccine uptake by country.

## Conclusions

In summary, we have found very high rates (almost 88%) of bacterial colonisation, viral infection and co-infections in hospital HCWs, far higher than rates previously described in adults. Most studies show that adults have rates much lower than children, yet the rates we demonstrated exceeded even colonisation rates in children, possibly reflecting hospitals being a high exposure setting. Respiratory tract infections were also dynamic and changing over time, with different HCWs infected at baseline and the end of the study, with different pathogens. We were unable to determine the relationship of symptoms to colonisation because of a small sample size, but suggest larger studies are warranted. Our results suggest there is a continual ongoing risk of respiratory infection in hospitals HCWs.

## Abbreviations

CDPC: Centre for Disease Prevention and Control
CRI: Clinical Respiratory Illness
HCW: Healthcare Worker
ICU: Intensive Care Unit
NAT: Nucleic acid testing
NP: Nasopharyngeal
PCR: Polymerase Chain Reaction
PPE: Personal Protective Equipment
